# Promises, Pitfalls, and Clinical Applications of Artificial Intelligence in Pediatrics

**DOI:** 10.2196/49022

**Published:** 2024-02-29

**Authors:** Hansa Bhargava, Carmela Salomon, Srinivasan Suresh, Anthony Chang, Rachel Kilian, Diana van Stijn, Albert Oriol, Daniel Low, Ashley Knebel, Sharief Taraman

**Affiliations:** 1 Children’s Hospital of Atlanta Atlanta, GA United States; 2 School of Medicine Emory University Atlanta, GA United States; 3 Healio South New Jersey, NJ United States; 4 Cognoa, Inc Palo Alto, CA United States; 5 Division of Health Informatics Department of Pediatrics University of Pittsburgh Pittsburgh, PA United States; 6 UPMC Children’s Hospital of Pittsburgh Pittsburgh, PA United States; 7 Fowler School of Engineering Chapman University Orange, CA United States; 8 SSI Strategy Parsippany, NJ United States; 9 Lapsi Health Amsterdam Netherlands; 10 Rady Children’s Hospital San Diego, CA United States; 11 AdaptX Seattle, WA United States; 12 Children’s Hospital of Orange County Orange, CA United States; 13 University of California Irvine School of Medicine Irvine, CA United States

**Keywords:** artificial intelligence, pediatrics, autism spectrum disorder, ASD, disparities, pediatric, youth, child, children, autism, autistic, barrier, barriers, clinical application, clinical applications, professional development, continuing education, continuing medical education, CME, implementation

## Abstract

Artificial intelligence (AI) broadly describes a branch of computer science focused on developing machines capable of performing tasks typically associated with human intelligence. Those who connect AI with the world of science fiction may meet its growing rise with hesitancy or outright skepticism. However, AI is becoming increasingly pervasive in our society, from algorithms helping to sift through airline fares to substituting words in emails and SMS text messages based on user choices. Data collection is ongoing and is being leveraged by software platforms to analyze patterns and make predictions across multiple industries. Health care is gradually becoming part of this technological transformation, as advancements in computational power and storage converge with the rapid expansion of digitized medical information. Given the growing and inevitable integration of AI into health care systems, it is our viewpoint that pediatricians urgently require training and orientation to the uses, promises, and pitfalls of AI in medicine. AI is unlikely to solve the full array of complex challenges confronting pediatricians today; however, if used responsibly, it holds great potential to improve many aspects of care for providers, children, and families. Our aim in this viewpoint is to provide clinicians with a targeted introduction to the field of AI in pediatrics, including key promises, pitfalls, and clinical applications, so they can play a more active role in shaping the future impact of AI in medicine.

## What is Artificial Intelligence in Medicine?

Artificial intelligence (AI) is a broad term describing the use of machine-based learning algorithms and software [1]. Nonmedical applications of AI include text prediction when we write emails or movie suggestions on cable and streaming based on our previous viewing choices. Machine learning (ML) is a specific branch of AI focused on altering programs or algorithms based on exposure to data in order to improve performance over time; in other words, the “machine” is “learning” as it accumulates more data and patterns. ML exists on a continuum. Supervised algorithms, for example, may require a great deal of outside input in order to function, while unsupervised algorithms can function with much greater degrees of autonomy [2]. One ML approach, useful when exploring complex nonlinear patterns, involves convolutional neural networks (CNNs). CNNs apply a special mathematical operation called a convolution across cumulative steps to perform specialized tasks like image processing. Natural language processing (NLP) solutions using ML are also being pioneered to help computers understand, categorize, and extract insights from natural language data sets [1]. In the field of medicine, AI has shown promise to assist with a wide array of clinical tasks, including risk prediction, diagnosis, and augmented decision-making and treatment and monitoring. Intelligent technology may also be leveraged to streamline workflows and automate some routine but historically time-intensive tasks, such as clinical note-taking [3]. NLP solutions, for example, have proven extremely useful in supporting clinicians to more efficiently leverage the large quantities of data collected in electronic medical records (EMRs) every day. NLP “reads” EMRs, attempting to understand medical words and phrases within the specific context of a given patient. IBM Watson, for example, has already successfully been used to examine large data sets of EMRs from diverse populations to create lists of common complications for a given population. Watson has also been used to gather medical literature in response to queries contained within examined EMRs [4].

In the following section of this viewpoint, we explore a selection of pediatric conditions, where AI-based approaches are being pioneered to various support risk prediction, diagnosis, therapy and response monitoring, or clinical workflow efficiency.

## AI in Pediatric Asthma Care

### Risk Prediction

Despite well-established clinical standards and pathways for asthma management, asthma remains one of the most frequent clinical concerns in pediatric offices and emergency rooms [5]. AI-based innovative solutions are being explored to help predict asthma risk so earlier intervention can occur. One study analyzed over 335,000 asthma events spanning the years 2005 to 2018 and then used ML to predict the risk of hospitalization for patients with asthma [6]. Similarly, another set of ML algorithms integrated clinical data with information on weather, neighborhood characteristics, and community viral load to predict the likelihood of hospitalization for children with asthma [7]. Another study used EHR data from 9934 children to train ML models to accurately predict the likelihood of a child’s asthma persisting over time [8].

### Monitoring and Medication Management

Several companies are pioneering efforts in this space. One company is optimizing AI for spirometry in a cell phone app [9]. The app captures a patient’s exhalation with the phone microphone and then translates these acoustic sounds into a curve representing lung volume. A portable spirometry test allows patients to conveniently track deterioration or therapeutic improvement over time. A separate mobile tool uses smart sensor data to register inhaler usage. This sensor analyzes the inhalation history of a patient in relation to weather conditions in order to recognize potential triggers of asthma attacks [10]. This analysis can also provide suggestions for the treatment dosage based on exacerbating factors present [10]. Another innovative digital therapy (DTx) uses AI to alert patients and parents to the early stages of an asthma attack. DTx continuously analyzes respiratory sounds in order to more quickly recognize changes in the sounds that could signal an oncoming asthmatic event. Ideally, warnings from DTx will help children and caregivers recognize the triggers as well as symptoms of an asthmatic crisis. Predicting outcomes in real time by using patient data and pattern detection algorithms patterns could lead to more precise and personalized asthma management [11].

## AI in Pediatric Rare Disease Management: Diagnostics

A leading children’s hospital has developed a program that uses pattern recognition and real-time data analysis to efficiently diagnose rare diseases in newborns. Leveraging AI to help automate genomic diagnosis could streamline and expedite the diagnostic process in neonatal intensive care units and pediatric intensive care units (PICUs). While clinical adoption considerations must first be addressed, such technology could potentially reduce delays to targeted life-changing treatments in newborns with diseases of previously unknown etiology [12].

## AI in Pediatric Sepsis: Risk Detection

Another serious concern in pediatrics is the early diagnosis of sepsis. In a study of almost 500 PICU patients, AI detected severe sepsis as early as 8 hours prior to traditional EMR-based screening algorithms [13]. This efficiency can have a profound impact on the management of sepsis in the PICU by allowing for earlier intervention and thus potentially reducing morbidity and mortality.

## AI and Opioid Dependence: Monitoring and Medication Management

In the United States, approximately 7% of adolescents develop persistent opioid use after surgery [14]. A new company focused on health and wellness for athletes recently made use of AI-powered software, OR Advisor (AdaptX). The use of OR Advisor enables frontline clinicians to perform rapid analysis of real-world data collected in their EMR. OR Advisor is also capable of tracking clinical metrics over time as well as monitoring treatment outcomes [14]. OR Advisor can help physicians more quickly answer clinically relevant questions, such as how adjusting medication doses might affect pain management. Using OR Advisor, Seattle Children’s Bellevue Clinic and Surgery Center was able to reduce opioid administration from 85% of surgeries to less than 1% [15]. Decreasing perioperative opioid use could potentially not only save thousands of lives per year but also reduce complications, thereby reducing the length of stay and improving recovery.

## AI in Radiology: Diagnostics

AI in radiology is perhaps the most impressive application of AI in medicine, with CNNs often performing as or even more accurately than clinicians. AI has already been applied in a variety of pediatric settings, such as scoliosis quantification, fracture and injury detection, and cystic fibrosis scoring on chest radiographs [16], but there are many other potential uses. One of the best potential uses of CNNs is as a screening tool for life-threatening diagnoses that need rapid intervention, such as acute ischemic strokes [17]. CNNs can accelerate the timeline of a non–life-threatening diagnosis (such as a small pneumonia) as well. Sophisticated AI image interpretation can also potentially lead to novel discoveries in medical images (such as tumor-stroma interfaces) that can help with subtyping, therapy modification, and prognostication of medical conditions [18]. To date, the majority of AI-based radiology solutions have been developed for adult populations. Radiology imaging advocacy groups have recently begun lobbying the US Congress to develop policies addressing the scarcity of pediatric-specific AI-based innovations [19].

## AI in Pediatric Autism: Diagnosis

AI is being leveraged to support improved behavioral and mental health outcomes in pediatric populations, including in children with autism. While autism diagnosis is possible as early as the age of 18 months, the average age of diagnosis in the United States remains older than 4 years [20,21]. The majority of these children are diagnosed in specialty care; however, the increasing demand for evaluations coupled with a shortage of specialists has contributed to lengthy waits for assessments [20,22,23]. Delays in treatment initiation can have lifelong implications for children and their families [24,25]. Applications using AI can potentially reduce these delays. For example, a Food and Drug Administration (FDA)–authorized AI-based diagnostic device, Canvas Dx, uses inputs from a child’s health care provider, caregiver, and video analysis to help health care providers rapidly diagnose or rule out autism in young children with concern for developmental delay [26-28]. The diagnostic device can be used in primary care settings, potentially decreasing delays to treatment initiation and thus significantly improving the child’s trajectory [27].

AI may also have a role to play in reducing diagnostic bias prevalent in some autism care pathways. Currently, compared to White peers, Black and Latino or Hispanic children receive later autism diagnoses [29,30]. One study found that in comparison to White children, Hispanic and Black children are 65% and 19% less likely to be diagnosed with autism and these racial or ethnic disparities exist regardless of economic class [29]. Similarly, girls are diagnosed with autism an average of 1.5 years later than their male peers [31,32]. Children from rural communities also experience lags in diagnosis and access issues. AI could help address some of these biases and promote diagnostic equity in several ways. Item-level racial or gender bias noted in some existing diagnostic and screening tools [33], for example, could be reduced through careful training of AI-based diagnostic alternatives on large samples of gender and racially diverse accurately labeled data. AI-based remotely administered technologies may also reduce access barriers for children in rural communities who face health care disparities due to geographical isolation, hospital closures, and insufficient clinical workforce coverage [34].

## Adolescent Depression and Anxiety: AI and Symptom Management

Mental health management in preteens and adolescents is another important concern in the primary care pediatric setting and can be especially challenging in the context of limited mental health resource availability. An estimated 31% of teens have an anxiety disorder [35]. The severity of these disorders and the scarcity of providers make it imperative that patients and caregivers have easily accessible resources that can be used while waiting to see a mental health provider. Such is the case with a research-based mental health software app, Woebot, that provides digital cognitive behavioral therapy to patients. Using AI, Woebot tailors its conversations to each patient, with the goal of providing personalized and high-quality care [33]. In a study published in *JMIR*, the intervention group experienced a 22% reduction in depressive symptoms over the course of 2 weeks after using this software in comparison with the control group [33]. The product was initially tested with subjects between the ages of 18 and 65 years; however, it may prove to be useful in the pediatric space as well, given ongoing mental health access and resource issues. Table S1 in Multimedia Appendix 1 [6-10,12,13,36,37] summarizes topical studies discussed in this viewpoint including further detail about methodology, performance metrics, and product development*.*

## Clinical Challenges and Concerns

Although AI has the potential to meaningfully benefit pediatric care, a number of regulatory, implementation, and ethical challenges exist. Additionally, poorly conceived AI has the potential to cause harm and therefore requires serious clinical consideration and risk identification and mitigation as part of any workflow implementation process. First, clinicians should be aware that training algorithms often require large data sets. In pediatrics, the small sample sizes of some data sets, especially when split by age group, can pose challenges when building unbiased AI algorithms. Relatedly, in order to build less biased algorithms, training data must be representative of the applicable populations across domains such as race, ethnicity, gender, socioeconomic status, and geographic contexts. Obtaining samples with known demographics and large enough subsamples of intersecting groups can prove challenging.

While we previously touched on the potential of AI to enhance health equity, the reverse may also be true. Without careful and equitable sampling, algorithms can potentially perpetuate rather than reduce bias. Even when this risk is mitigated by training AI algorithms on a diverse patient base, bias can still occur at the data labeling level. This is because the algorithm may be “learning” from the implicit biases of humans who are annotating the input data or validating its output classifications. Clinicians can help mitigate these risks by requesting information on the size and diversity of the training data used and through careful interrogation of any potential labeling biases.

Also considering that even when AI is built on representative data sets and yields generalizable algorithms, implementation challenges may reduce the extent to which such technologies can address inequities in practice. An unbiased algorithm implemented inequitably could, in fact, maintain or perpetuate disparities in pediatric health care. Families without access to a computer or reliable internet connectivity, for example, may not be able to access some digital technologies that could benefit them. As AI-powered tools become more commonplace in clinical practice, we recommend that their implementation be accompanied by physician education, antibias training, and system-wide change aimed at making health care more accessible to marginalized populations. Similarly, disparities in access to technology should be addressed. These goals should be supported through tactics such as the development of consensus ethical frameworks for the use of AI health care, the introduction of robust AI and ethics curriculum in medical education, as well as regulatory frameworks.

There are a number of ethical questions to consider when integrating AI-based technologies into clinical pathways. For example, what are the implications of physicians relying on outputs from complex, multidimensional ML models in clinical decision-making when they may have limited visibility into how such models arrive at diagnostic conclusions? [3,38] Thoughtful use of technology also necessitates weighing the benefits of intelligent technology against the potential scale of harm that could occur if a widely deployed algorithm were to malfunction [3]. Open questions also remain about who should be held accountable for incorrect diagnostic or treatment recommendations produced by devices powered by AI [39]. Some patients have also raised concerns about how their data privacy and security will be maintained within such systems [40]. In response to these questions, a number of ethical frameworks for the use of AI in clinical practice are being developed [41,42]. These include action points related to building institutional capabilities to redress any emerging AI-related harms; distinguishing between appropriate and inappropriate AI task delegation; developing metrics to help measure and monitor the trustworthiness of AI-based technologies; and financially incentivizing the inclusion of ethical, legal, and social considerations in AI research projects [41].

Open questions also remain about how to effectively regulate AI-based medical technologies. To date, the FDA has cleared more than 160 AI-powered devices for use [43]. Existing regulatory frameworks, however, do not account for the ability of intelligent devices to iteratively “learn” from the data they are exposed to and alter their algorithms in response [44]. Should such features be disabled in approved products to avoid drift in performance and function? If not, how should such features be scrutinized and regulated on an ongoing basis? The FDA proposed regulatory framework for software as medical device technologies provide some direction for potential solutions such as predetermined change control plan and algorithm change plan protocol specifications [45].

## Future of AI in Medical Education

As clinical AI apps become more commonplace, pediatricians will require fundamental training and orientation to the data science behind AI so they can use AI-enabled tools appropriately and evaluate their strengths and weaknesses. However, AI integration into medical curriculum is currently limited and inconsistent, and no topical content is tested in key licensing examinations [46]. This has left both students and practicing clinicians with knowledge and confidence gaps regarding the application of AI in clinical practice [47-49].

Medical schools should establish introductory AI courses to foster clinical confidence and competence. The future pediatrician will benefit greatly from the synergy between AI and human cognition. A number of proposed curriculum updates are being explored [46,50,51], including MEd2030, an updated medical school curriculum that will add 10 additional modules (eg, biomedical informatics and AI, transdisciplinary collaboration and diversity principles, biomedical entrepreneurship, design innovation) [52]. In the continuing education sector, conferences and programs on digital therapeutics and AI-based health technologies are also growing as more companies introduce FDA-regulated products.

Actively engaging with AI-based technologies will allow pediatricians to play a greater role in the implementation and use of AI in every aspect of health care. Learning to apply developing technologies is one key to optimizing patient care and improving the future of health care systems globally. When EMRs were first introduced in the 2000s, many clinicians knew about them, but few were engaged in the implementation and continual improvement of the technology. EMRs have since infiltrated almost every aspect of a clinician’s practice; yet, as a result of limited early-stage clinical engagement, they are often not optimized for the clinical workflow [1]. By seeking to understand and engage with the next wave of technological advances earlier and more proactively, pediatricians can help ensure they will be more tailored to the needs of both care providers and patients.

## Conclusions

Health care delivery models are changing rapidly, and AI is poised to greatly impact the future of pediatric care. Efficient diagnostic tools, faster data analysis, predictive outcomes modeling, more streamlined care experiences, and automation of some previously time-intensive tasks are just some of the potential benefits offered by AI. However, widespread clinical integration of AI into pediatric care pathways will also require thoughtful solutions to complex data quality and privacy issues, and ethical and regulatory implementation barriers. We recommend that tailored AI-focused curriculum and ongoing training opportunities be developed and implemented rapidly to support pediatricians and medical students alike to responsibly use and understand the strengths and limitations of such technologies more deeply. With improved education, we are optimistic about the future potential of AI to enhance clinical efficiencies and outcomes and to support broader access to high-quality pediatric health care in marginalized communities.

## Appendix

Multimedia Appendix 1 Summary of reviewed studies.

## Abbreviations

AI: artificial intelligence
CNN: convolutional neural network
DTx: digital therapy
EMR: electronic medical record
FDA: Food and Drug Administration
ML: machine learning
NLP: natural language processing
PICU: pediatric intensive care unit
