# Supplementary material for: Promises, Pitfalls, and Clinical Applications of Artificial Intelligence in Pediatrics
Source: J Med Internet Res. 2024 Feb 29;26:e49022. doi: 10.2196/49022 (PMC10940991; doi:10.2196/49022)
Supplement: Multimedia Appendix 1 [file jmir_v26i1e49022_app1.docx]

**Table S1**. Clinical AI applications.

| Condition | Application | References | Performance and considerations |
| --- | --- | --- | --- |
| Asthma | Machine learning classification to predict hospitalization for asthma patients. | Luo et al [6] | The model achieved an AUC of 0.859 (95% CI 0.846-0.871). When the cutoff threshold for conducting binary classification was set at the top 10.00% (1926/19,256) of asthmatic patients with the highest predicted risk, the model reached an accuracy of 90.31% (17,391/19,256; 95% CI 89.86-90.70), a sensitivity of 53.7% (436/812; 95% CI 50.12-57.18), and a specificity of 91.93% (16,955/18,444; 95% CI 91.54-92.31). |
|  |  | Patel et al [7] | The AUCs for each model were: decision tree 0.72 (95% CI 0.66–0.77), logistic regression 0.83 (95% CI 0.82–0.83), random forests 0.82 (95% CI 0.81–0.83), and gradient boosting machines 0.84 (95% CI 0.83–0.85). |
| Asthma | Machine learning to identify  which children diagnosed with asthma before age 5 continue to experience persistent asthma. | Bose et al [8] | All studied models performed significantly better than random chance, with XGBoost obtaining the best performance (0.43 mean ANSA). Feature importance analysis indicated age of last asthma diagnosis under 5 years, total number of asthma related visits, self-identified black race, allergic rhinitis, and eczema as important features. |
| Asthma | Portable spirometry test. | Larson et al [9] | When evaluated in 52 subjects the mean error when compared to a clinical spirometer was 5.1% for common measures of lung function. |
| Asthma | A.I.-based tool that monitors use of an inhaler and provides patient-specific suggestions for treatment. | Van Sickle et al [10] | The intervention group demonstrated significant improvements compared to the control group on all clinical outcomes, including controller medication adherence, daily SABA use, asthma-free days, and asthma control (all p<0.001), including a 21-point improvement in adherence for the intervention group. |
| Rare Diseases | AI to help automate genomic diagnosis helping diagnose rare diseases in newborns | Clark et al [12] | Automated, retrospective diagnoses concurred well with expert manual interpretation (97% recall and 99% precision in 95 children with 97 genetic diseases). Prospectively, the platform correctly diagnosed three of seven seriously ill ICU infants (100% precision and recall) with a mean time saving of 22:19 hours. In each case, the diagnosis affected treatment. |
| Sepsis | Artificial intelligence used to predict the  onset of severe sepsis using physiologic data for critically ill children in the PICU | Kamaleswaran et al [13] | When analyzing the physiomarkers present in the 2–8 hours analysis window, logistic regression performed with specificity of 87.4% and sensitivity of 55.0%, random forest performed with 79.6% specificity and 80.0% sensitivity, and the Convolutional Neural Network performed with 83.0% specificity and 75.0% sensitivity. When analyzing physiomarkers from the 8–24 hours window, logistic regression resulted in 77.1% specificity and 39.3% sensitivity, random forest performed with 82.3% specificity and 61.1% sensitivity, whereas the Convolutional Neural Network method achieved 81% specificity and 76% sensitivity. |
| Scoliosis | Deep learning artificial intelligence algorithms applied to images of children’s backs accurately identified scoliosis presence and severity. | Yang et al [36] | The accuracies of the algorithms was higher than the human specialists in detecting scoliosis, detecting cases with a curve ≥20°, and severity grading for both binary classifications and the four-class classification. |
| Autism Spectrum Disorder | Machine learning enabled software that analyzes inputs from health care providers and caregivers to diagnose or rule out autism in 18-72 month olds. | Wall et al [37] | The device, with optimized decision thresholds, produced a determinate (yes/no for autism) result for 66.5% (95% CI, 62.5–71.0) of children in the study. PPV and NPV were 87.5% (95% CI, 82.5–96.7) and 95.6% (95% CI, 93.7–97.9) respectively. |
| Anxiety /Depression | Mental health software application that provides digital Cognitive Behavior Therapy to the patient. | Prochaska et al [33] | From pre- to posttreatment, confidence to resist urges to use substances significantly increased (mean score change +16.9, SD 21.4; P<.001), whereas past month substance use occasions (mean change −9.3, SD 14.1; P<.001) and scores on the Alcohol Use Disorders Identification Test-Concise (mean change −1.3, SD 2.6; P<.001), 10-item Drug Abuse Screening Test (mean change −1.2, SD 2.0; P<.001), Patient Health Questionnaire-8 item (mean change 2.1, SD 5.2; P=.005), Generalized Anxiety Disorder-7 (mean change −2.3, SD 4.7; P=.001), and cravings scale (68.6% vs 47.1% moderate to extreme; P=.01) significantly decreased. |
| Surgery | AI-enabled software to leverage EMR data to perform opioid-free outpatient surgery. | Low [15] | Example: over the course of 14 months, post-operative morphine requirements dropped from 22% to 13% of patients. Pain scores and the 30-day return to surgery rate did not increase. |
